# Electroencephalography for prognostication of outcome in adults with severe herpes simplex encephalitis

**DOI:** 10.1186/s13613-023-01110-3

**Published:** 2023-02-23

**Authors:** Lina Jeantin, Claire Dupuis, Geoffroy Vellieux, Pierre Jaquet, Etienne de Montmollin, Jean-François Timsit, Romain Sonneville, the ENCEPHALITICA Study Group, Mikael Alves, Laurent Argaud, Pierre Bailly, François Barbier, Lila Bouadma, Noelle Brulé, Fabrice Bruneel, Russell Chabanne, Marie Conrad, Daniel da Silva, Frederic Dailler, Delphine Daubin, Sophie Demeret, Nicolas Lerolle, Julien Marechal, Bruno Mourvillier, Ahmed El Kalioubi, Benjamine Sarton, Stein Silva, Vincent Susset, Jean Marc Tadié, Jean-Françoit Timsit, Michel Wolff, Alexandre Lautrette, Emmanuel Novy, Bertrand Guidet, François Mateos, Clément Brault, Quentin Maestraggi, Keyvan Razazi, Jean-Pierre Quenot, Aurélie Joret, Albrice Levrat, Alexandre Massri, Alexandre Robert, Damien Contou, Jean-Paul Mira, Gaudry Stephane, Guillaume Voiriot, Asael Berger, Vincent Das, Nicolas Engrand, Martin Murgier, Shidasp Siami, Sami Hraiech, Eric Mariotte, Claire Ragot, Annabelle Stoclin, Pierre Trouiller, Mathieu Schmidt, Charline Sazio

**Affiliations:** 1 Department of Neurology, GHU Paris Psychiatrie et Neurosciences, Université de Paris, Paris, France; 2 Department of Intensive Care Medicine, Clermont-Ferrand University Hospital, 63000 Clermont-Ferrand, France; 3 Paris Brain Institute, ICM, Inserm, CNRS, Sorbonne Université, 75013 Paris, France; 4 Department of Neurophysiology, Pitie-Salpêtrière University Hospital, AP-HP, Paris, France; 5 Department of Intensive Care Medicine, Delafontaine Hospital, Saint Denis, France; 6 IAME, INSERM, UMR1137, Université Paris Cité, Paris, France; 7 Department of Intensive Care Medicine, Bichat-Claude Bernard University Hospital, AP-HP, 46 Rue Henri Huchard, 75877 Paris Cedex, France

**Keywords:** Herpes simplex, Encephalitis, Electroencephalography, Prognosis, Critical care outcomes, Functional status

## Abstract

**Background:**

Electroencephalography (EEG) is recommended for the practical approach to the diagnosis and prognosis of encephalitis. We aimed to investigate the prognostic value of standard EEG (_std_EEG) in adult patients with severe herpes simplex encephalitis.

**Methods:**

We performed a retrospective analysis of consecutive ICU patients with severe herpes simplex encephalitis in 38 French centers between 2006 and 2016. Patients with at least one _std_EEG study performed at ICU admission were included. _std_EEG findings were reviewed independently by two investigators. The association between _std_EEG findings (i.e., background activity, lateralized periodic discharges, seizures/status epilepticus, and reactivity to painful/auditory stimuli) and poor functional outcome, defined by a score on the modified Rankin Scale (mRS) of 3 to 6 (moderate to severe disability or death) at 90 days, were investigated.

**Results:**

We included 214 patients with at least one available _std_EEG study. The first _std_EEG was performed after a median time of one (interquartile range (IQR) 0 to 2) day from ICU admission. At the time of recording, 138 (64.5%) patients were under invasive mechanical ventilation. Lateralized periodic discharges were recorded in 91 (42.5%) patients, seizures in 21 (9.8%) and status epilepticus in 16 (7.5%). In the whole population, reactivity to auditory/noxious stimuli was tested in 140/214 (65.4%) patients and was absent in 71/140 (33.2%) cases. In mechanically ventilated patients, _std_EEG reactivity was tested in 91/138 (65.9%) subjects, and was absent in 53/91 (58.2%) cases. Absence of reactivity was the only independent _std_EEG finding associated with poor functional outcome in the whole population (OR 2.80, 95% CI 1.19 to 6.58) and in the subgroup of mechanically ventilated patients (OR 4.99, 95% CI 1.6 to 15.59). Adjusted analyses for common clinical predictors of outcome and sedation at time of _std_EEG revealed similar findings in the whole population (OR 2.03, 95% CI 1.18 to 3.49) and in mechanically ventilated patients (OR 2.62, 95% CI 1.25 to 5.50).

**Conclusions:**

Absence of EEG reactivity to auditory/noxious stimuli is an independent marker of poor functional outcome in severe herpes simplex encephalitis.

**Supplementary Information:**

The online version contains supplementary material available at 10.1186/s13613-023-01110-3.

## Background

Herpes simplex virus (HSV) is the leading cause of sporadic encephalitis in adult patients [1]. It is a life-threatening disease with a mortality rate of 6–10% [2], which can rise to 70% without appropriate treatment [3, 4]. However, despite early initiation of intravenous acyclovir, HSV encephalitis (HSE) remains associated with a poor neurological outcome, as half of the patients present moderate to severe disability at 1 year [5, 6]. The practical diagnostic approach to suspected HSE encephalitis requires an early multimodal evaluation including a clinical examination, brain imaging, HSV DNA detection in the cerebrospinal fluid (CSF) by polymerase-chain reaction (PCR), and standard electroencephalography (_std_EEG) [7].

Standard EEG is often considered as an easily accessible, non-specific tool in the intensive care unit (ICU). It is recommended for initial evaluation of patients with suspected encephalitis to detect focal or diffuse changes suggestive of encephalitis and to rule out non-convulsive seizures [7]. Standard EEG patterns during HSE include changes in background activity in 46.4% of cases, focal or generalized slowing in 28.6%, seizures or status epilepticus in 17.8%, and periodic discharges in 11.5% [8–12]. However, the prognostic value of _std_EEG changes in HSE has been little studied. In a single-center retrospective study conducted on 103 patients hospitalized for all-cause encephalitis, including 12 HSE, a normal _std_EEG was independently associated with a lower relative risk of death [12]. In another retrospective series of 45 HSE, _std_EEG abnormalities were not associated with functional outcome [13]. A retrospective cohort study of 42 patients with primary central nervous system (CNS) infections, including 27 viral infections, found that continuous EEG monitoring recorded seizures in about 33% of patients and periodic epileptiform discharges in 40%, both independently associated with poor outcome [14]. However, continuous EEG monitoring data in primary CNS infections has been scarcely reported.

In the present study, we aimed to investigate the prognostic value of common _std_EEG findings observed in severe adult cases of HSE encephalitis requiring care in the ICU.

## Methods

### Design

We conducted a retrospective analysis of patients from the multicenter HERPETICS database, which included adult patients with HSV encephalitis between January 2006 and December 2016 in 38 ICUs in France [15]. The ethical committee of the French society of Intensive Care Medicine (FICS) approved the study and waived the requirement for informed consent. Inclusion criteria were: (a) a minimum age of 18 years; (b) admission to the ICU with possible or probable acute encephalitis, as defined by the Consensus Statement of the International Encephalitis Consortium; [7] (c) a positive CSF PCR for HSV DNA during hospitalization; (d) and at least one _std_EEG recording performed at admission or during ICU stay. Exclusion criteria were: (a) missing data on clinical outcome at 90 days; (b) missing or incomplete _std_EEG data. Standard EEG data was reviewed independently by 2 investigators, including a neurologist (LJ) and a neurophysiologist (GV).

### Clinical biological and brain imaging data

We used the data previously collected for the HERPETICS database, including patients’ history, neurological symptoms and reason for ICU admission, clinical, biological and imaging data, and therapeutics [15].

### Standard EEG studies

We collected the following data from _std_EEG reports: date of study, indication, and ongoing sedative and/or antiseizure medication at the time of the recording. We followed the American Clinical Neurophysiology Society’s guidelines to describe findings collected from _std_EEG reports [16]: background activity voltage, asymmetry, continuity and frequency (minimal and maximal), periodic discharges, interictal epileptic activities (e.g. sharp waves, spikes, spike-and-waves), electrographic or electroclinical seizures, status epilepticus, and reactivity to external stimuli. A background frequency in the alpha range is defined by a frequency > 7 Hz but < 13 Hz [16]. Status epilepticus was defined as an electroclinical seizure for ≥ 10 continuous minutes or for a total duration of ≥ 20% of any 60-min period of recording, or ≥ 5 continuous minutes in cases of a bilateral tonic–clonic activity [16]. Reactivity was defined as transient changes in the _std_EEG voltage and/or frequency immediately after an auditory or noxious stimulus. Reactivity on _std_EEG recordings was categorized as “present”, “absent”, or “not tested” (when no auditory/noxious stimulus was applied during the recording) [16].

### Second _std_EEG

If more than one _std_EEG study was available, we collected data from the first and second recordings. We studied evolution of reactivity between subsequent _std_EEG studies and classified patients as “preserved reactivity” when both _std_EEG were reactive to external stimuli, “absent reactivity” when none of the two _std_EEG was reactive, and “variable reactivity” in patients with discordant reactivity between _std_EEG studies (presence of reactivity followed by absence of reactivity, or vice versa).

### Outcomes

The primary outcome was a poor functional outcome at 90 days, defined by a score on the modified Rankin Scale (mRS) of 3 to 6, indicating moderate to severe disability or death.

### Statistical analysis

Patients’ characteristics are expressed as counts and frequencies for categorical variables and medians (interquartile range, IQR) for quantitative variables. Comparisons were achieved using the Wilcoxon rank sum test for continuous variables and Fisher’s test for categorical variables. We investigated the association of the first _std_EEG findings with poor functional outcome in the whole cohort by means of univariable and multivariable logistical regression analyses. First, variables associated with outcome in univariable analysis (p-value < 0.20) were entered in the multivariable model. Second, we applied a backward elimination to select variables for the final model. Secondary analyses included a subgroup analysis in patients under mechanical ventilation and adjusted analyses for common clinical predictors of outcome (i.e., age, coma, and body temperature) and sedation at the time of EEG recording. Missing data were treated with multiple imputations using PROC MI and PROC MIANALYZE (SAS Software). All tests were two-sided and a p-value < 0.05 was considered statistically significant. All analyses were performed using SAS software, version 9.4 (SAS Institute, Cary, NC) and R statistical software version 3.5.2 (R Project for statistical computing).

## Results

A total of 286 patients were screened, and 214 patients from 38 centers were included in the study (see patients flow chart, Additional file 1: Figure S1). There was no difference between characteristics of included patients who benefited from a _std_EEG, and those of excluded patients who did not have a _std_EEG recording or had missing _std_EEG data (Additional file 1: Table S1). Baseline characteristics of patients are detailed in Table 1. The median age was 63 years (IQR 53–72) and 111 (51.9%) patients were male. A total of 105 (49.1%) patients were admitted to the ICU for altered mental status and 54 (25.2%) patients for seizures. 138 (64.5%) patients underwent mechanical ventilation.

**Table 1:** Patients’ characteristics and EEG description at ICU admission

Variables	Total n = 214	mRS 0–2 n = 57	mRS 3–6 n = 157	p-value
**Patients**				
Age (years)	63 [53; 72]	57.4 [44.4; 69.1]	65.4 [56.3; 75.4]	< 0.01
Male sex	111 (51.9)	30 (52.6)	81 (51.6)	0.89
Reason for ICU admission				
Altered mental status	105 (49.1)	26 (45.6)	79 (50.3)	0.11
Seizure	54 (25.2)	20 (35.1)	34 (21.7)	
Other*	55 (25.7)	11 (19.3)	44 (28)	
Glasgow coma scale^¤^				
Score	9 [6; 12]	10 [7; 13]	8.5 [6; 12]	0.06
< 8, indicating coma	73/203 (36)	16/55 (29.1)	57/148 (38.5)	0.21
Temperature^§^				
Degrees (°C)	38.8 [38.1; 39.2]	38.5 [38; 39]	39 [38.2; 39.2]	0.07
≥ 38.3 °C, indicating fever	142/201 (70.6)	33/53 (62.3)	109/148 (73.6)	0.12
Focal signs	33/211 (15.6)	12/57 (21.1)	21/154 (13.6)	0.19
**CSF**				
HSV 1 genotype^∇^	175/183 (95.6)	44/47 (93.6)	131/136 (96.3)	0.43
Leukocytes (/mm^3^)^$^	51 [12; 150]	100 [31; 200]	38.5 [9; 125]	0.01
Lymphocytes (%)^∃^	88 [61; 96]	85.4 [36.5; 205.8]	69.7 [25.2; 270]	0.37
Protein level (g/l)^⊥^	0.7 [0.5; 1.1]	0.7 [0.5; 1]	0.7 [0.5; 1.2]	0.85
Glycorachia^⊥⊥^	4 [3.2; 4.5]	3.9 [3.4; 4.6]	4 [3; 4.4]	0.20
Hypoglycorachia^⊥⊥^	18/165 (10.9)	4/46 (8.7)	14/119 (11.8)	0.66
Abnormal MRI at admission	188/190 (98.9)	53/55 (96.4)	135/135 (100)	0.02
Invasive mechanical ventilation	138 (64.5)	28 (49.1)	110 (70.1)	< 0.01
**EEG**				
Time between ICU admission and _std_EEG (in days)	1 [0; 2]	1 [0; 1]	1 [0; 2]	0.16
EEG recorded with sedation	81/211 (88.4)	20/56 (35.7)	61/155 (39.4)	0.63
Background rhythm				
Low voltage^†^	32/209 (15.3)	11/55 (20)	21/154 (13.6)	0.26
Asymmetry in voltage	25/213 (11.7)	4/57 (7)	21/156 (13.5)	0.20
Asymmetry in frequency	65/213 (30.5)	17/56 (30.4)	48/157 (30.6)	0.98
Discontinuous rhythm^‡^	21/213 (9.9)	5/57 (8.8)	16/57 (28)	0.79
Maximal background frequency recorded				
Alpha (> 7 Hz)	60 (28)	23 (40.4)	37 (23.6)	0.05
Frequency not specified	45 (21)	11 (19.3)	34 (21.7)	
< 7 Hz	109 (50.9)	23 (40.4)	86 (54.8)	
Minimal background frequency recorded				
Alpha (> 7 Hz)	23 (10.7)	10 (17.5)	13 (8.3)	0.12
Frequency not specified	52 (24.3)	11 (19.3)	41 (26.1)	
< 7 Hz	139 (65)	36 (63.2)	103 (65.6)	
Presence of lateralized periodic discharges (LPDs)	91 (42.5)	26 (45.6)	65 (41.4)	0.58
Interictal epileptic activities				
Spikes or sharps	46 (21.5)	12 (21.1)	34 (21.7)	0.92
Spike-and-wave/sharp-and-wave	14 (6.5)	2 (3.5)	12 (7.6)	0.28
Seizures	21 (9.8)	5 (8.8)	16 (10.2)	0.76
Status epilepticus	16 (7.4)	2 (3.5)	14 (8.9)	0.18
Reactivity				
Present	69 (32.2)	24 (42.1)	45 (28.7)	0.01
Not tested	74 (34.6)	23 (40.4)	51 (32.5)	
Absent	71 (33.2)	10 (17.5)	61 (38.9)	

Results expressed as medians [quartiles] or numbers (%)

Attenuation: periods ≥ 10 µV but < 50% of the higher voltage background

Suppression: periods of lower voltage are < 10 µV of the higher voltage background

mRS: modified Rankin Scale; ICU: intensive care unit; _std_EEG: standard electroencephalography; GCS: Glasgow Coma Scale; CSF: cerebrospinal fluid; HSV: herpes simplex virus; MRI: magnetic resonance imaging

^ϕ^A good functional status prior to admission was defined by a Knaus score of A or B

^£^Initial admission to hospital wards vs direct ICU admission

^*^Other reasons included mainly respiratory failure

^¤^GCS was determined in 203 patients

^§^Temperature was determined in 201 patients

^∇^Data about HSV genotype was available for 183 out of 214 patients (others had unspecified HSV positivity in the CSF)

^$^Leukocyte count was determined in 205 patients

^∃^Lymphocyte count was determined in 133 patients

^⊥^Protein level was determined in 183 patients

^⊥⊥^Glycorachia level was estimated in 165 patients

^†^Includes low voltage (i.e. 10–20 µV) and suppressed voltage (i.e. < 10 µV)

^‡^Includes discontinuous (10–49% attenuated or suppressed), burst attenuation/suppression (50–99% attenuated or suppressed), suppression/attenuation (> 99% attenuated or suppressed)

**Fig. 1:**
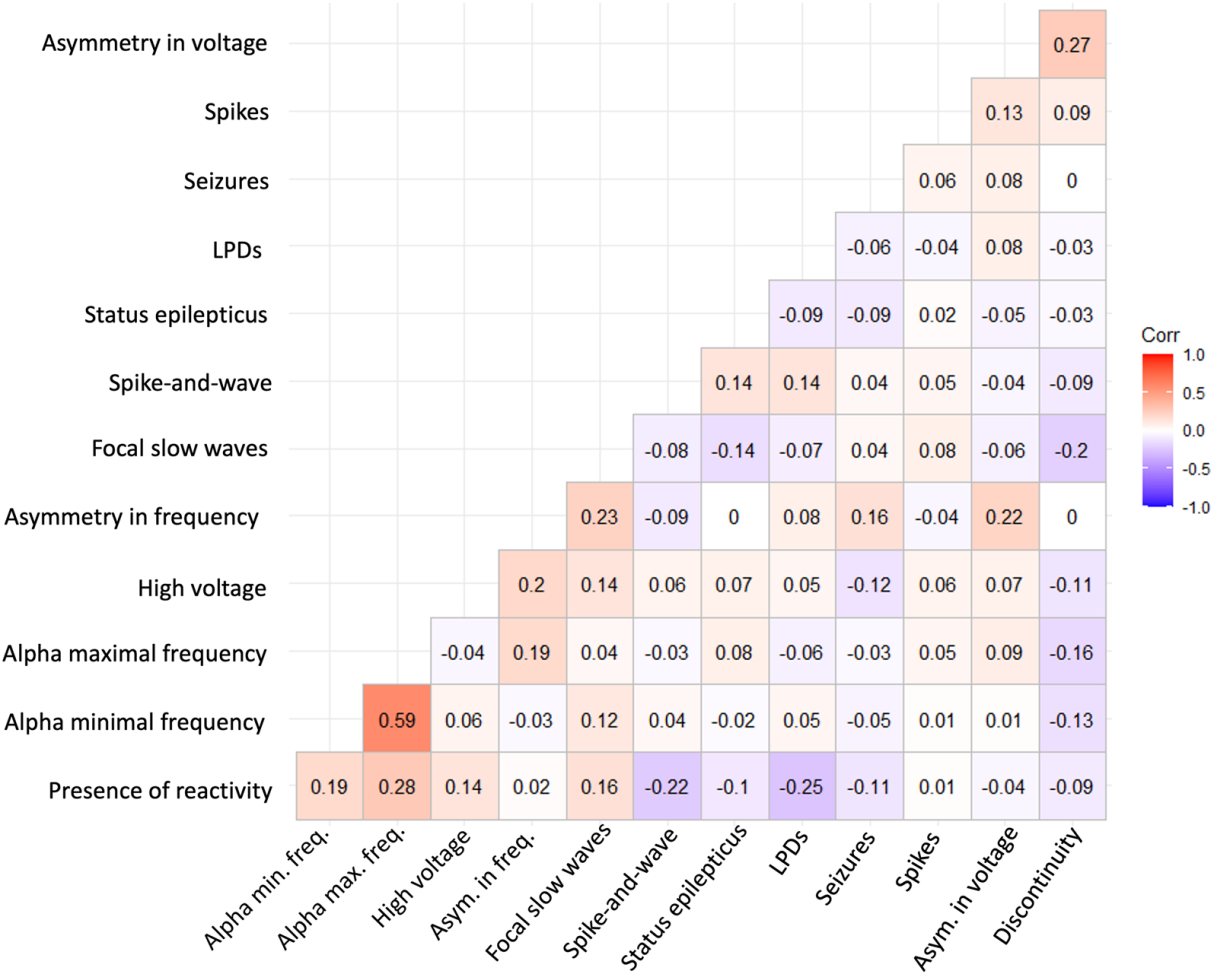
Heat map of correlation coefficient values between EEG variables. The correlation coefficients are represented on a colored scale from − 1.0 to 1.0. Discontinuity: discontinuous background rhythm; LPDs: Lateralized periodic discharges

Among the 214 patients, 140 (65.4%) subjects had one _std_EEG, 42 (19.6%) had two _std_EEGs, 14 (6.5%) had 3 and 18 (8.4%) had more than 3 _std_EEGs. Data from the first available _std_EEG is detailed in Table 1 and the correlation between EEG variables at the time of the recording is represented in Fig. 1. Median delays between ICU admission and the first _std_EEG and between acyclovir administration and the first _std_EEG were 1 (IQR 0–2) day, and 1 (IQR 1–3) day, respectively. Twenty-seven patients had their first _std_EEG before admission to the ICU (with a median delay of 2 days (IQR 1–5) for these patients). Out of 192 _std_EEG studies, 105 (54.7%) were performed for suspicion of encephalitis, 79/192 (41.1%) to rule out seizures and status epilepticus and 16/192 (8.3%) for both of these motives. Thirty-two patients out of 209 (15.3%) _std_EEG recordings presented with a suppressed or low voltage, 25/213 (11.7%) were asymmetric in voltage and 65 (30.5%) were asymmetric in frequency. Thirteen out of 169 (7.7%) recordings had a maximal background rhythm frequency in the delta range. Lateralized periodic discharges (LPDs) were recorded in 91 (42.5%) patients. Sixty (28%) patients had interictal epileptic activities with spikes or sharp waves in 46 (21.5%) patients and spike-and-waves in 14 (6.5%) patients. Seizures were recorded in 21 (9.8%) patients and 16 (7.5%) presented with status epilepticus at the time of the first _std_EEG. Reactivity was tested in 140/214 (65.4%) patients and was present in 69/140 (49.3%) recordings. No differences were found between the characteristics of patients for whom reactivity was tested, and those for whom it was not studied (Additional file 1: Table S1).

Overall, 157 (73.4%) patients had a poor functional outcome at 90 days, including 38 (17.8%) deaths. Results of the multivariable analyses of factors associated with poor functional outcome are presented in Table 2, and detail for the univariable and multivariable analyses can be found in Additional file 1: Table S2. The characteristics of _std_EEG included in the multivariable model were maximal background frequency, and _std_EEG reactivity. A _std_EEG with “absent reactivity” was found to be the only variable independently associated with poor functional outcome (OR 2.80, 95% CI 1.19 to 6.58). A maximal background frequency recorded in the alpha range was found not to be independently associated with functional outcome (p = 0.187) in the multivariable analysis (Additional file 1: Table S2).

**Table 2:** Association of _std_EEG reactivity with poor functional outcome, multivariable analyses

	Model including EEG variables		Model including clinical and EEG variables	
	Adjusted odds ratio*	95% CI	Adjusted odds ratio**	95% CI
**Whole cohort (n = 214)**				
Present reactivity	1	–	1	–
Reactivity not tested	1.11	0.53–2.30	0.73	0.45–1.20
Absent reactivity	2.80	1.19–6.58	2.03	1.18–3.49
**Patients under mechanical ventilation (n = 138)**				
Present reactivity	1	–	1	–
Reactivity not tested	1.92	0.73–5.07	1.12	0.57–2.20
Absent reactivity	4.99	1.60–15.59	2.62	1.25–5.50

^*^Odds ratios presented in the model including EEG variables are adjusted for maximal background frequency

^**^Odds ratios presented in the model including clinical and EEG variables are adjusted for maximal background frequency, age, Glasgow coma score < 8, temperature ≥ 38.3 °C, time between ICU admission and _std_EEG > 1 day, and the presence of sedation at the time of EEG recording. The AUC [95%CI] of the final model was 0.764 [95% CI, 0.759 to 0.769]

A secondary subgroup analysis performed in patients under mechanical ventilation (Table 2 and Additional file 1: Table S3) confirmed that absence of _std_EEG reactivity was significantly associated with poor functional outcome in this population (OR 4.99, 95% CI 1.6 to 15.59). Secondary adjusted analyses for common clinical predictors of outcome and sedation at time of EEG revealed similar findings in the whole population (OR 2.03, 95% CI 1.18 to 3.49) and in patients under mechanical ventilation (OR 2.62, 95% CI 1.25 to 5.50).These analyses are presented in Table 2, Additional file 1: Tables S2bis and S3bis.

**Fig. 2:**
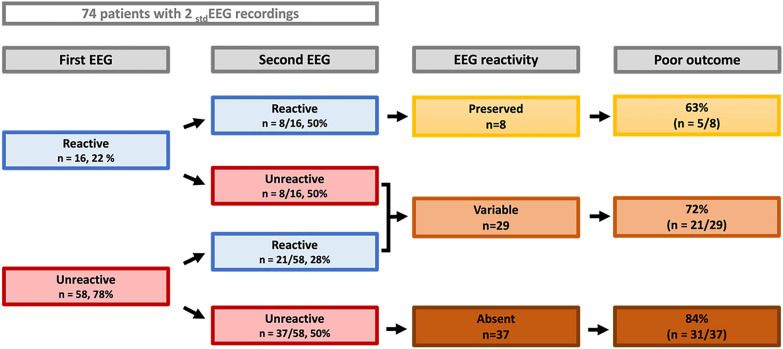
Association of EEG reactivity on the first and second _std_EEGs with outcome. If more than one _std_EEG study was available, we collected data from the first and second studies. We classified patients as “preserved reactivity” when both _std_EEG were reactive to external stimuli, “absent reactivity” when none of the two _std_EEG was reactive, and “variable reactivity” in patients with discordant reactivity between _std_EEG studies (presence of reactivity followed by absence of reactivity, or vice versa). A poor outcome was defined by a score of 3–6 on the modified Rankin scale

Seventy-four (35%) patients had at least a second _std_EEG recording after a median time of 5 (IQR 2–12) days from the ICU admission. As for the first recording, the subsequent _std_EEG were performed for seizures in 37/71 (52.1%) patients, for encephalitis suspicion in 14/71 (19.7%) patients, for both of these motives in 1/71 (1.4%) patients or to control the abnormalities shown on the first _std_EEG in 16/71 (22.5%) patients. Sixteen (22%) patients had a first _std_EEG with “present reactivity” and eight (50%) of them presented with preserved reactivity on the second _std_EEG (Fig. 2). Of these eight patients with preserved reactivity in both _std_EEGs, 5 (63%) had poor functional outcome at day 90. On the contrary, 58 (78%) patients had a first _std_EEG with absence of reactivity, and among them 37 (50%) had a second _std_EEG where reactivity was still absent. A persistent _std_EEG with absent reactivity was associated with a poor functional outcome in 31/37 (84%) of these patients. Twenty-nine patients, classified as “variable reactivity”, had discordant reactivity between _std_EEGs, and 21/29 (72%) patients had a poor functional outcome.

## Discussion

This retrospective study assembled 214 critically ill patients with HSE from 38 centers, with available _std_EEG data and assessment of poor functional outcome (i.e., moderate to severe disability or death) at day 90. Our findings suggest that the lack of EEG reactivity to external stimuli in critically ill patients with HSE is independently associated with poor functional outcome. This result was confirmed in a subgroup analysis of mechanically ventilated patients, who can be considered as the most severely affected patients with HSE encephalitis. Moreover, the prognostic value of absent EEG reactivity was independent of common prognostic factors identified in this population (including age, coma, and fever at admission) [15] and was independent of sedation at time of EEG recording. Previous monocentric studies conducted in the non-ICU setting identified EEG patterns associated with outcome [8], such as Synek grades of I and II being predictive of good neurological prognosis. To our knowledge, our study is the largest cohort of patients with herpes simplex encephalitis requiring care in an ICU investigating _std_EEG patterns associated with poor neurological prognosis.

The results from our study add to the recent body of evidence highlighting the prognostic role of EEG reactivity in critically ill patients with primary or secondary brain injury [17–19]. These results are consistent with a recent retrospective study of 121 unresponsive ICU patients with various diagnoses (e.g., respiratory, circulatory or neurologic failure), showing that the combination of the presence of reactivity on a _std_EEG with a background frequency greater than 4 Hz was the only variable independently associated with a reduced risk of death [20]. Another study combined the same markers for assessing prognosis, and showed that a lower background frequency was independently associated with unfavorable outcome at 28 days in patients under venoarterial extracorporeal membrane oxygenation [21]. In our study, we could not easily combine markers such as background frequency and reactivity since minimal or maximal background frequencies were not reinterpreted as continuous variables and were not retained in the final multivariable model.

Standard EEG is of crucial importance for the positive diagnosis of herpes simplex encephalitis and its complications, notably seizures, status epilepticus and periodic discharges that can indicate parenchymal necrosis. Previous studies have mainly investigated the role of _std_EEG in the positive diagnosis of HSE, especially in complex cases [11], and little data is available about prognostication. A retrospective, monocentric cohort of 29 patients, with _std_EEG recordings performed in 25 patients, found _std_EEG patterns (i.e. Synek III to V) to be associated with poor clinical outcome at 6 months [8]. However, _std_EEG data only included Synek grade and the presence of LPDs, and reactivity to external stimuli was not reported.

The prognostic value of _std_EEG recordings, however, has been studied in brain injuries of other kinds. In a large registry of cardiac arrest patients, absence of EEG reactivity was predictive of unfavorable outcome [18]. Preserved EEG reactivity was found to be associated with good clinical outcome after cardiac arrest in a post-hoc analysis of a prospective study, especially in patients with a discontinuous normal voltage _std_EEG background pattern [22]. A recent review emphasized the link between preserved _std_EEG reactivity and favorable clinical outcomes in consciousness impairments of various etiologies, but highlights the need for homogenous and consensual methods for reactivity assessment during _std_EEG recordings [17].

Interestingly, the persistence of reactivity over time could be associated with neurological outcome. Indeed, when dividing the 74 patients who had at least two _std_EEGs during hospitalization into three groups, we noted a worsening of the neurological prognosis when _std_EEGs reactivity remained absent over time. Patients who had reactivity present on both _std_EEGs (“preserved reactivity” group) had poor neurological prognosis in 63% of cases. Those who showed reactivity on neither _std_EEG (“absent reactivity” group) had a poor prognosis in 84% of cases. The intermediate group (“uncertain reactivity”) with reactivity being present in only one of the two _std_EEGs, seemed to evolve towards a poor prognosis in 72% of cases. Although our data was underpowered to allow proper analysis given the small number of patients who benefited from two _std_EEGs or more, our study suggests that repeating _std_EEG recordings as a follow-up for reactivity could be helpful during the ICU stay. This result requires confirmation by a prospective study with a standardized protocol (e.g. performing a _std_EEG upon arrival to the ICU, followed by recordings at regular intervals).

Clinical elements for prognostication in HSE have been identified in previous studies, such as older age, coma, admission body temperature and indirect admission to an intensive care unit [15]. Our study did not find seizures or status epilepticus to be associated with poor clinical outcome in HSE. A retrospective study of 54 critical care unit patients with continuous _std_EEG monitoring diagnosed with viral or auto-immune encephalitis found 22 (41%) patients who presented seizures [23]. Patients with seizures had significantly more LPDs, low voltage and focal slowing, but seizures were not associated with functional outcome at discharge, in accord with the present results. However, only 11 patients had HSV encephalitis and 12 had encephalitis of unknown etiology.

Since only two patients had a normal MRI at ICU admission in our cohort, MRI lesions were already present in the majority at the time of _std_EEGs recording. Our data was therefore insufficient to determine whether _std_EEG could show abnormalities before MRI signs can be detected. A recent multicenter cohort study investigated MRI data associated with poor functional outcome and found that MRI signal abnormalities involving more than 3 cerebral lobes were associated with poor functional prognosis [24]. Lateralized periodic discharges are often considered as an EEG marker of brain injury. However, in our study, we did not find the number of cerebral lobes impaired by lateralized periodic discharges to be associated with a poorer neurological prognosis. This could be explained by the poor spatial resolution of _std_EEG, making the precise localization of lesions and their extension difficult to establish.

The strength of this study was to assemble a large multicenter cohort of 214 HSE patients recruited from 38 ICUs in reference centers and smaller hospitals, which decreases the risk of center bias. The large number of patients allowed to performed multivariable analyses adjusted for common clinical predictors of outcome in this population. Our study also has limitations: firstly, this study had a retrospective design, which implies missing data (e.g. missing _std_EEG reports) and lack of reproducibility between subjects: some patients had a _std_EEG upon arrival to the ICU, others were recorded a few days later for complications or delay in the recovery of consciousness. This retrospective design did not allow proper standardization of _std_EEG recordings, or a systematic assessment of reactivity with a unique protocol (same stimuli and recording length). Many _std_EEG reports reported scarce data, not always in accordance with the American Clinical Neurophysiology Society’s standardized critical care EEG terminology [16], which might have resulted in the loss of important information. For instance, assessing Synek grades, a scale using _std_EEG patterns to establish prognosis in adult patients with diffuse anoxic and traumatic encephalopathies [25], was only possible for a small portion of individuals in our study. Analyzing full _std_EEG tracings by two independent reviewers, instead of _std_EEG reports of various origins, could be a way to determine _std_EEG patterns in HSE in a more objective way, but such tracings were unfortunately unavailable for retrieval. Moreover, the reports did not mention detailed clinical data at the time of the _std_EEG recording, such as Glasgow Coma Scale, FOUR score or response to command, and most of the _std_EEG reports had no precisions about the duration of the recording and number of electrodes. For the patients under mechanical ventilation, we could find no precision about the presence and type of sedation at the time of the recording. Secondly, our study lacks continuous _std_EEG recordings which could allow a better monitoring of neurophysiological status (e.g., evolution and duration of status epilepticus or seizures), as some brief epileptic events might not be recorded with discontinuous short-lasting _std_EEGs. This might have underpowered our study in assessing neurological prognosis and mortality in some patients, especially for epileptic complications. Thirdly, our population was limited to patients from intensive care units, which makes it difficult to extrapolate our results to less severely affected populations, such as patients admitted to neurology or infectious diseases departments. Finally, we observed that out of the 286 patients of the Herpetics database, 13 had not benefited from a _std_EEG recording, while electroencephalography is recommenced for any suspicion of encephalitis. This could be explained by the large amount of centers in the study, including smaller hospitals, with no on-site Neurophysiology department and a more difficult access to electroencephalography, reflecting real-life access to the usual standard of care.

A prospective study with a standardized protocol for the timing of _std_EEG recordings and the assessment of reactivity would be useful to extend our results. The persistence of _std_EEG abnormalities over time (e.g. lack of reactivity) could also be studied, rather than the immediate _std_EEG abnormalities in the acute phase. Such a study would allow the assessment of long-term cognitive prognosis, quality of life and epilepsy sequelae. However, HSE cases remain scarce, making the construction of such a protocol difficult.

## Conclusion

The absence of electroencephalographical reactivity to external stimuli is an independent indicator of poor functional outcome in adult patients with severe herpes simplex encephalitis, notably for mechanically ventilated patients. In addition to its use for the diagnosis of herpes simplex encephalitis and its complications, electroencephalography could help identify prognostic factors in often complex clinical situations.

## Supplementary Information

**Additional file 1: Figure S1.** Patients flow chart. **Table S1.** Patients’ characteristics, according to the inclusion/exclusion in the study, and according to the testing of reactivity. **Table S2.** Results of the uni- and multivariable analyses for all patients (n = 214). **Table S2bis.** Results of the uni- and multivariable analyses for all patients, including clinical data and adjusted for sedation (n = 214). **Table S3.** Results of the uni- and multivariable analyses for patients under mechanical ventilation (n = 138). **Table S3bis.** Results of the uni- and multivariable analyses for patients under mechanical ventilation, including clinical data and adjusted for sedation (n = 138). **Table S4.** Complete case analysis for the whole cohort (n = 194), and among patients under mechanical ventilation (n = 122).

## Data Availability

The datasets used and analyzed during the current study are available from the corresponding author on reasonable request.
