# Supplementary material for: Electroencephalography for prognostication of outcome in adults with severe herpes simplex encephalitis
Source: Ann Intensive Care. 2023 Feb 23;13:10. doi: 10.1186/s13613-023-01110-3 (PMC9950306; doi:10.1186/s13613-023-01110-3)
Supplement: Supplementary file 1 — Additional file 1: Figure S1. Patients flow chart. Table S1. Patients’ characteristics, according to the inclusion/exclusion in the study, and according to the testing of reactivity. Table S2. Results of the uni- and multivariable analyses for all patients (n = 214). Table S2bis. Results of the uni- and multivariable analyses for all patients, including clinical data and adjusted for sedation (n = 214). Table S3. Results of the uni- and multivariable analyses for patients under mechanical ventilation (n = 138). Table S3bis. Results of the uni- and multivariable analyses for patients under mechanical ventilation, including clinical data and adjusted for sedation (n = 138). Table S4. Complete case analysis for the whole cohort (n = 194), and among patients under mechanical ventilation (n = 122). [file 13613_2023_1110_MOESM1_ESM.docx]

**Supplementary Figure 1:** Patients flow chart

**Patients flow chart**

**Records not included** (n = 13):

No EEG performed during hospital stay (n = 13)

**Inclusion in the HERPETICS database** (n = 286):

(1) Admitted to the ICU with possible acute encephalitis (2) Positive CSF PCR for HSV

**Identification**

**Records screened for EEG neuroprognostication**

(n = 273)

**Records excluded** (n = 59):

Missing or incomplete EEG report (n = 32)

Missing outcome data (n = 27)

**Screening**

**Inclusion in the study** (n = 214)

**Included**

**Supplementary Table 1:** Patients’ characteristics, according to the inclusion/exclusion in the study, and according to the testing of reactivity.

|  | **Inclusion/exclusion of the study** | | | **Testing of reactivity** | | |
| --- | --- | --- | --- | --- | --- | --- |
| **Variables** | **Excluded**  n = 72 | **Included**  n = 214 | **p-value** | **Reactivity tested**  n = 140 | **Reactivity not tested**  n = 74 | **p-value** |
| **Age** (years) | 64.8 [58.7 ; 74.6] | 63.8 [53 ; 72.9] | 0.48 | 64.5 [54.5 ; 72.6] | 62.2 [51.5 ; 76.1] | 0.91 |
| < 50 | 16 (22.2) | 45 (21) | 0.99 | 25 (17.9) | 20 (27) | 0.10 |
| ≥ 50 and < 65 | 14 (19.4) | 41 (19.2) | . | 26 (18.6) | 15 (20.3) | . |
| ≥ 65 and < 75 | 23 (31.9) | 73 (34.1) | . | 46 (32.9) | 27 (36.5) | . |
| ≥ 75 | 19 (26.4) | 55 (25.7) | . | 43 (30.7) | 12 (16.2) | . |
| **Male sex** | 34 (47.2) | 111 (51.9) | 0.50 | 73 (52.1) | 38 (51.4) | 0.91 |
| **Coexisting conditions** |  |  |  |  |  |  |
| Immunocompromised | 11 (15.3) | 42 (19.6) | 0.41 | 22 (15.7) | 20 (27) | 0.05 |
| Diabetes | 8/68 (11.8) | 30 (14) | 0.64 | 21 (15) | 9 (12.2) | 0.57 |
| Alcohol abuse | 10/68 (14.7) | 28 (13.1) | 0.73 | 20 (14.3) | 8 (10.8) | 0.47 |
| Psychiatric disease | 5/68 (7.4) | 20 (9.3) | 0.61 | 16 (11.4) | 4 (5.4) | 0.15 |
| Epilepsy | 2/69 (2.9) | 9 (4.2) | 0.63 | 6 (4.3) | 3 (4.1) | 0.94 |
| **Functional status before admission** |  |  |  |  |  |  |
| Knaus score A or B ^φ^ | 67/69 (97.1) | 202 (94.4) | 0.37 | 133 (95) | 69 (93.2) | 0.60 |
| mRS ≥ 3 | 3/69 (4.3) | 11 (5.1) | 0.79 | 7 (5) | 4 (5.4) | 0.90 |
| **Admission from another ward** ^£^ | 25/71 (35.2) | 104/212 (49.1) | 0.04 | 68/139 (48.9) | 36/73 (49.3) | 0.96 |
| **Reason for ICU admission** |  |  |  |  |  |  |
| Altered mental status | 35 (48.6) | 105 (49.1) | 0.89 | 74 (52.9) | 31 (41.9) | 0.17 |
| Seizure | 20 (27.8) | 54 (25.2) | . | 30 (21.4) | 24 (32.4) | . |
| Other * | 17 (23.6) | 55 (25.7) | . | 36 (25.7) | 19 (25.7) | . |
| **Glasgow coma scale** ^¤^ |  |  |  |  |  |  |
| Score | 8 [6 ; 12] | 9 [6 ; 12] | 0.54 | 9 [6 ; 12] | 8.5 [4 ; 13] | 0.77 |
| < 8, indicating coma | 26/63 (41.3) | 73/203 (36) | 0.45 | 46/133 (34.6) | 27/70 (38.6) | 0.57 |
| **Temperature** ^§^ |  |  |  |  |  |  |
| Degrees (°C) | 38.5 [38 ; 39.3] | 38.8 [38.1 ; 39.2] | 0.52 | 38.8 [38.2 ; 39.2] | 38.8 [38.1 ; 39.1] | 0.77 |
| ≥38.3°C, indicating fever | 39/62 (62.9) | 142/201 (70.6) | 0.25 | 96/133 (72.2) | 46/68 (67.6) | 0.50 |
| **Convulsive seizures at admission** |  |  |  |  |  |  |
| Seizure(s) | 17 (23.6) | 54 (25.2) | 0.74 | 36 (25.7) | 18 (24.3) | 0.31 |
| Status epilepticus | 9 (12.5) | 20 (9.3) | . | 16 (11.4) | 4 (5.4) | . |
| **Focal signs** | 12/68(17.65) | 33/212(15.57) | 0.75 | 21 (15) | 12/72 (16.7) | 0.76 |
| **CSF** |  |  |  |  |  |  |
| HSV 1 genotype ^∇^ | 47/50 (94) | 175/183 (95.6) | 0.63 | 113/120 (94.2) | 62/63 (98.4) | 0.18 |
| Leukocytes (/mm^3^) ^$^ | 37.5 [7 ; 116] | 51 [12 ; 150] | 0.37 | 48.5 [13 ; 145] | 55 [10 ; 160] | 0.80 |
| Lymphocytes (%) ^∃^ | 84.8 [36.5 ; 256] | 74.4 [27 ; 223.6] | 0.92 | 72 [25.3 ; 198.3] | 81.7 [29.1 ; 264.9] | 0.62 |
| Protein level (g/l) ^⊥^ | 0.7 [0.6 ; 1.3] | 0.7 [0.5 ; 1.1] | 0.16 | 0.7 [0.5 ; 1.2] | 0.6 [0.5 ; 1] | 0.25 |
| Glycorrhachia (mmol/L) ^⊥⊥^ | 3.9 [3.3 ; 4.7] | 4 [3.2 ; 4.5] | 0.45 | 3.8 [3 ; 4.3] | 4.2 [3.5 ; 5] | 0.01 |
| Hypoglycorrhachia | 5/45 (11.1) | 18/165 (10.9) | 0.97 | 14/110 (12.7) | 4/55 (7.3) | 0.29 |
| **Brain imaging** |  |  |  |  |  |  |
| Abnormal CT-scan | 17/56 (30.4) | 64/181 (35.4) | 0.49 | 40/118 (33.9) | 24/63 (38.1) | 0.57 |
| Normal MRI | 3/56 (5.4) | 2/190 (1.1) | 0.04 | 1/127 (0.8) | 1/63 (1.6) | 0.61 |
| Lesions > 3 lobes on the MRI | 4/56 (7.1) | 62/190 (32.6) | <0.01 | 38/127 (29.9) | 24/63 (38.1) | 0.26 |
| **Initial management** |  |  |  |  |  |  |
| Time between onset of symptoms and ICU admission (days) ^#^ | 3 [1 ; 5] | 4 [2 ; 6] | 0.05 | 3 [2 ; 5.5] | 4 [2 ; 6] | 0.65 |
| Time between ICU admission and initiation of acyclovir (days) ^##^ | 0 [-1 ; 0] | 0 [-1 ; 0] | 0.62 | 0 [-1 ; 0] | 0 [-1 ; 0] | 0.20 |
| **Invasive mechanical ventilation** | 42 (58.3) | 138 (64.5) | 0.35 | 91 (65) | 47 (63.5) | 0.83 |

Results expressed as median [quartiles] or numbers (%)

*Abbreviations: mRS: modified Rankin Scale; ICU: intensive care unit; GCS: Glasgow Coma Scale; CSF: cerebrospinal fluid; HSV: herpes simplex virus; CT: computed tomography; MRI: magnetic resonance imaging*

^φ^ A good functional status prior to admission was defined by a Knaus score of A or B

^£^ Initial admission to hospital wards vs direct ICU admission

* Other reasons included mainly respiratory failure.

For the included/excluded analysis (left part of the table) and for the reactivity tested/not tested analysis (right part of the table), respectively:

^¤^ GCS was determined in 266 and 203 patients.

^§^ Temperature was determined in 263 and 201 patients.

^∇^ Data about HSV genotype was available for 233 out of 286 patients (other had unspecified HSV positivity in the CSF) and for 183 out of 214 patients.

^$^ Leukocyte count was determined in 267 and 205 patients.

^∃^ Lymphocyte count was determined in 164 and 133 patients.

^⊥^ Protein level was determined in 239 and 183 patients.

^⊥⊥^ Glycorrhachia was determined in 210 and 165 patients.

^#^ Time between onset of symptoms and ICU admission was determined in 251 and 189 patients.

^##^ Time between ICU admission and initiation of acyclovir was determined in 281 and 212 patients.

**Supplementary Table 2:** Results of the uni- and multivariable analyses for all patients (n = 214)

|  | **Univariable (BI)** | | **Univariable (AI)** | | **Multivariable (AI)** | |
| --- | --- | --- | --- | --- | --- | --- |
|  | OR [95 CI] | p-value | OR [95 CI] | p-value | OR [95 CI] | p-value |
| **Maximal background frequency recorded** |  |  |  |  |  |  |
| Alpha (> 7Hz) | 1.00 | 0.05 | 1.00 | 0.05 | 1 | 0.187 |
| Background frequency not specified | 1.92 [0.82 ; 4.52] | 0.55 | 1.92 [0.82 ; 4.52] | 0.55 | 1.66 [0.67 ; 4.09] | 0.663 |
| < 7Hz | 2.32 [1.16 ; 4.66] | 0.11 | 2.32 [1.16 ; 4.66] | 0.11 | 1.93 [0.94 ; 3.95] | 0.221 |
| **Minimal background frequency recorded** |  |  |  |  |  |  |
| Alpha (> 7Hz) | 1.00 | 0.13 | 1.00 | 0.13 |  |  |
| Background frequency not specified | 2.87 [0.99 ; 8.27] | 0.11 | 2.87 [0.99 ; 8.27] | 0.11 |  |  |
| < 7Hz | 2.20 [0.89 ; 5.45] | 0.43 | 2.20 [0.89 ; 5.45] | 0.43 |  |  |
| **Low voltage of background rhythm** | 0.63 [0.28 ; 1.41] | 0.26 | 0.65 [0.29; 1.46] | 0.30 |  |  |
| **Discontinuous background rhythm** | 1.19 [0.41 ; 3.41] | 0.75 | 1.25 [0.44; 3.56] | 0.68 |  |  |
| **Asymmetry in voltage** | 2.06 [0.68 ; 6.29] | 0.20 | 2.11 [0.69; 6.44] | 0.19 |  |  |
| **Asymmetry in frequency** | 1.01 [0.52 ; 1.96] | 0.98 | 0.99 [0.51; 1.91] | 0.97 |  |  |
| **Reactivity** |  |  |  |  |  |  |
| Present | 1.00 | 0.02 | 1.00 | 0.02 | 1 | 0.043 |
| Not tested | 1.18 [0.59 ; 2.38] | 0.20 | 1.18 [0.59 ; 2.38] | 0.20 | 1.11 [0.53 ; 2.3] | 0.225 |
| Absent | 3.25 [1.42 ; 7.48] | <0.01 | 3.25 [1.42 ; 7.48] | <0.01 | 2.80 [1.19 ; 6.58] | 0.013 |
| **Epileptiform discharges** |  |  |  |  |  |  |
| Spike | 1.04 [0.49 ; 2.18] | 0.92 | 1.04 [0.49 ; 2.18] | 0.92 |  |  |
| Spike-and-wave | 2.28 [0.49 ; 10.5] | 0.29 | 2.28 [0.49 ; 10.5] | 0.29 |  |  |
| **Presence of LPD** | 0.84 [0.46 ; 1.55] | 0.58 | 0.84 [0.46 ; 1.55] | 0.58 |  |  |
| **Seizure** | 1.18 [0.41 ; 3.38] | 0.76 | 1.18 [0.41 ; 3.38] | 0.76 |  |  |
| **Status epilepticus** | 2.69 [0.59 ; 12.23] | 0.20 | 2.69 [0.59 ; 12.23] | 0.20 |  |  |

Results are expressed as OR [95 CI]. Abbreviations: AI: after imputation (multiple imputations were used to adjust for missing values), BI: before imputation, Hz: Hertz, LPD: Lateralized periodic discharges

**Supplementary table 2bis:** Results of the uni- and multivariable analyses for all patients, including clinical data and adjusted for sedation (n = 214)

|  | **Univariable (BI)** | | **Univariable (AI)** | | **Multivariable (AI)** | |
| --- | --- | --- | --- | --- | --- | --- |
| **Variable** | OR [95 CI] | p-value | OR [95 CI] | p-value | OR [95 CI] | p-value |
| **Age** (years) |  |  |  |  |  |  |
| < 50 | 1.00 | <.01 | 1.00 | <.01 | 1 |  |
| ≥ 50 and < 65 | 2.22 [0.99 ; 4.99] | 0.75 | 2.22 [0.99 ; 4.99] | 0.75 | 1.06 [0.61; 1.83] | 0.84 |
| ≥ 65 and < 75 | 1.91 [0.82 ; 4.45] | 0.40 | 1.91 [0.82 ; 4.45] | 0.40 | 0.66 [0.36; 1.20] | 0.17 |
| ≥ 75 | **8.02 [2.42 ; 26.57]** | **<.01** | **8.02 [2.42 ; 26.57]** | **<.01** | **3.27 [1.40; 7.63]** | **<0.01** |
| **Glasgow coma scale < 8,** indicating coma | 1.53 [0.78 ; 2.98] | 0.22 | 1.50 [0.77; 2.93] | 0.23 | 1.23 [0.82; 1.81] | 0.30 |
| **Temperature ≥38.3°C**, indicating fever | 1.69 [0.87 ; 3.29] | 0.12 | 1.69 [0.87; 3.26] | 0.12 | 1.40 [0.97; 2.01] | 0.07 |
| **Time between ICU admission and _std_EEG > 1 day** | **2.59 [1.24 ; 5.38]** | **0.01** | **2.59 [1.24 ; 5.38]** | **0.01** | **1.74 [1.15; 2.62]** | **<0.01** |
| **Maximal background frequency recorded** |  |  |  |  |  |  |
| Alpha (> 7Hz) | 1.00 | 0.05 | 1.00 | 0.05 |  |  |
| Background frequency not specified | 1.92 [0.82 ; 4.52] | 0.55 | 1.92 [0.82 ; 4.52] | 0.55 | 1.26 [0.71; 2.25] | 0.44 |
| < 7Hz | 2.32 [1.16 ; 4.66] | 0.11 | 2.32 [1.16 ; 4.66] | 0.11 | 1.12 [0.70 ; 1.81] | 0.63 |
| **Reactivity** |  |  |  |  |  |  |
| Present | 1.00 | 0.02 | 1.00 | 0.02 |  |  |
| Not tested | 1.18 [0.59 ; 2.38] | 0.20 | 1.18 [0.59 ; 2.38] | 0.20 | 0.73 [0.45; 1.2] | 0.21 |
| Absent | **3.25 [1.42 ; 7.48]** | **<.01** | **3.25 [1.42 ; 7.48]** | **<.01** | **2.03 [1.18. 3.49]** | **0.01** |
| **Minimal background frequency recorded** |  |  |  |  |  |  |
| Alpha (> 7Hz) | 1.00 | 0.13 | 1.00 | 0.13 |  |  |
| Background frequency not specified | 2.87 [0.99 ; 8.27] | 0.11 | 2.87 [0.99 ; 8.27] | 0.11 |  |  |
| < 7Hz | 2.20 [0.89 ; 5.45] | 0.43 | 2.20 [0.89 ; 5.45] | 0.43 |  |  |
| **Low voltage of background rhythm** | 0.63 [0.28 ; 1.41] | 0.26 | 0.65 [0.29; 1.46] | 0.30 |  |  |
| **Discontinuous background rhythm** | 1.19 [0.41 ; 3.41] | 0.75 | 1.25 [0.44; 3.56] | 0.68 |  |  |
| **Asymmetry in voltage** | 2.06 [0.68 ; 6.29] | 0.20 | 2.11 [0.69; 6.44] | 0.19 |  |  |
| **Asymmetry in frequency** | 1.01 [0.52 ; 1.96] | 0.98 | 0.99 [0.51; 1.91] | 0.97 |  |  |
| **Epileptiform discharges** |  |  |  |  |  |  |
| Spike | 1.04 [0.49 ; 2.18] | 0.92 | 1.04 [0.49 ; 2.18] | 0.92 |  |  |
| Spike-and-wave | 2.28 [0.49 ; 10.5] | 0.29 | 2.28 [0.49 ; 10.5] | 0.29 |  |  |
| **Presence of LPD** | 0.84 [0.46 ; 1.55] | 0.58 | 0.84 [0.46 ; 1.55] | 0.58 |  |  |
| **Seizure** | 1.18 [0.41 ; 3.38] | 0.76 | 1.18 [0.41 ; 3.38] | 0.76 |  |  |
| **Status epilepticus** | 2.69 [0.59 ; 12.23] | 0.20 | 2.69 [0.59 ; 12.23] | 0.20 |  |  |
| **Presence of sedation** | 1.16 [1.05 ; 1.28] | <0.01 | 1.07 [0.93 ; 1.47] | 0.65 | 0.91 [0.63 ; 1.33] | 0.64 |

Results are expressed as OR [95 CI]. Abbreviations: AI: after imputation (multiple imputations were used to adjust for missing values), BI: before imputation, Hz: Hertz, LPD: Lateralized periodic discharges

**Supplementary Table 3:** Results of the uni- and multivariable analyses for patients under mechanical ventilation (n = 138)

|  | **Univariable (BI)** | | **Univariable (AI)** | | **Multivariable (AI)** | |
| --- | --- | --- | --- | --- | --- | --- |
|  | OR | p-value | OR | p-value | OR | p-value |
| **Maximal background rhythm** |  |  |  |  |  |  |
| Alpha (> 7Hz) | 1.00 | 0.55 | 1.00 | 0.55 |  |  |
| Background frequency not specified | 1.38 [0.43 ; 4.48] | 0.92 | 1.38 [0.43 ; 4.48] | 0.92 |  |  |
| < 7Hz | 1.72 [0.65 ; 4.52] | 0.38 | 1.72 [0.65 ; 4.52] | 0.38 |  |  |
| **Minimal background rhythm** |  |  |  |  |  |  |
| Alpha (> 7Hz) | 1.00 | 0.21 | 1.00 | 0.21 |  |  |
| Background frequency not specified | 2.60 [0.68 ; 9.99] | 0.41 | 2.60 [0.68 ; 9.99] | 0.41 |  |  |
| < 7Hz | 2.78 [0.88 ; 8.74] | 0.22 | 2.78 [0.88 ; 8.74] | 0.22 |  |  |
| **Low voltage of background rhythm** | 0.50 [0.17 ; 1.45] | 0.20 | 0.55 [0.19; 1.6] | 0.27 |  |  |
| **Discontinuous background rhythm** | 0.74 [0.22 ; 2.51] | 0.63 | 0.79 [0.24; 2.65] | 0.7 |  |  |
| **Asymmetry in voltage** | 2.40 [0.52 ; 11.08] | 0.26 | 2.48 [0.54; 11.4] | 0.24 |  |  |
| **Asymmetry in frequency** | 1.70 [0.63 ; 4.59] | 0.29 | 1.58 [0.59; 4.23] | 0.36 |  |  |
| **Reactivity** |  |  |  |  |  |  |
| Reactivity | **1** | **0.02** | **1** | **0.02** | **1** | **0.02** |
| Reactivity not tested | 1.92 [0.73 ; 5.07] | 0.75 | 1.92 [0.73 ; 5.07] | 0.75 | 1.92 [0.73 ; 5.07] | 0.75 |
| No Reactivity | **4.99 [1.6 ; 15.59]** | **0.02** | **4.99 [1.6 ; 15.59]** | **0.02** | **4.99 [1.6 ; 15.59]** | **0.02** |
| **Epileptiform discharges** |  |  |  |  |  |  |
| Spike | 1.25 [0.46 ; 3.4] | 0.66 | 1.25 [0.46 ; 3.4] | 0.66 |  |  |
| Spike-and-wave | - | - | - | - |  |  |
| **Presence of LPD** | 0.99 [0.43 ; 2.3] | 0.99 | 0.99 [0.43 ; 2.3] | 0.99 |  |  |
| **Seizure** | 1.02 [0.27 ; 3.89] | 0.98 | 1.02 [0.27 ; 3.89] | 0.98 |  |  |
| **Status epilepticus** | 3.30 [0.41 ; 26.53] | 0.26 | 3.30 [0.41 ; 26.53] | 0.26 |  |  |

Results are expressed as OR [95 CI]. Abbreviations: AI: after imputation (multiple imputations were used to adjust for missing values), BI: before imputation, Hz: Hertz, LPD: Lateralized periodic discharges

**Supplementary table 3bis**: Results of the uni- and multivariable analyses for patients under mechanical ventilation, including clinical data and adjusted for sedation (n = 138)

|  | **Univariable (BI)** | | **Univariable (AI)** | | **Multivariable (AI)** | |
| --- | --- | --- | --- | --- | --- | --- |
| **Variable** | OR [95 CI] | p-value | OR [95 CI] | p-value | OR [95 CI] | p-value |
| **Age** (years) |  |  |  |  |  |  |
| < 50 |  | 0.20 |  | 0.20 |  |  |
| ≥ 50 and < 65 | 1.80 [0.57 ; 5.66] | 0.73 | 1.80 [0.57 ; 5.66] | 0.73 | 0.98 [0.47; 2.05] | 0.96 |
| ≥ 65 and < 75 | 1.81 [0.55 ; 6.01] | 0.76 | 1.81 [0.55 ; 6.01] | 0.76 | 1.04 [0.46; 2.39] | 0.91 |
| ≥ 75 | **5.17 [1.16 ; 22.97]** | **0.05** | **5.17 [1.16 ; 22.97]** | **0.05** | 2.29 [0.83; 6.35] | 0.11 |
| **Glasgow coma scale < 8,** indicating coma | 1.10 [0.47 ; 2.56] | 0.83 | 1.06 [0.45; 2.48] | 0.89 | 1.14 [0.69; 1.87] | 0.62 |
| **Temperature ≥38.3°C**, indicating fever | 1.83 [0.74 ; 4.55] | 0.19 | 1.80 [0.73; 4.45] | 0.20 | 1.49 [0.88; 2.51] | 0.13 |
| **Time between ICU admission and _std_EEG > 1 day** | **3.04 [0.98 ; 9.41]** | **0.05** | **3.04 [0.98 ; 9.41]** | **0.05** | **5.47 [1.49; 20.05]** | **0.01** |
| **Maximal background frequency recorded** |  |  |  |  |  |  |
| Alpha (> 7Hz) | 1.00 | 0.55 | 1.00 | 0.55 |  |  |
| Background frequency not specified | 1.38 [0.43 ; 4.48] | 0.92 | 1.38 [0.43 ; 4.48] | 0.92 |  |  |
| < 7Hz | 1.72 [0.65 ; 4.52] | 0.38 | 1.72 [0.65 ; 4.52] | 0.38 |  |  |
| **Reactivity** |  |  |  |  |  |  |
| Present | **1** | **0.02** | **1** | **0.02** |  |  |
| Not tested | 1.92 [0.73 ; 5.07] | 0.75 | 1.92 [0.73 ; 5.07] | 0.75 | 1.12 [0.57; 2.20] | 0.75 |
| Absent | **4.99 [1.6 ; 15.59]** | **0.02** | **4.99 [1.6 ; 15.59]** | **0.02** | **2.62 [1.25; 5.50]** | **0.02** |
| **Minimal background frequency recorded** |  |  |  |  |  |  |
| Alpha (> 7Hz) | 1.00 | 0.21 | 1.00 | 0.21 |  |  |
| Background frequency not specified | 2.60 [0.68 ; 9.99] | 0.41 | 2.60 [0.68 ; 9.99] | 0.41 |  |  |
| < 7Hz | 2.78 [0.88 ; 8.74] | 0.22 | 2.78 [0.88 ; 8.74] | 0.22 |  |  |
| **Low voltage of background rhythm** | 0.50 [0.17 ; 1.45] | 0.20 | 0.55 [0.19; 1.6] | 0.27 |  |  |
| **Discontinuous background rhythm** | 0.74 [0.22 ; 2.51] | 0.63 | 0.79 [0.24; 2.65] | 0.7 |  |  |
| **Asymmetry in voltage** | 2.40 [0.52 ; 11.08] | 0.26 | 2.48 [0.54; 11.4] | 0.24 |  |  |
| **Asymmetry in frequency** | 1.70 [0.63 ; 4.59] | 0.29 | 1.58 [0.59; 4.23] | 0.36 |  |  |
| **Epileptiform discharges** |  |  |  |  |  |  |
| Spike | 1.25 [0.46 ; 3.4] | 0.66 | 1.25 [0.46 ; 3.4] | 0.66 |  |  |
| Spike-and-wave | - | - | - | - |  |  |
| **Presence of LPD** | 0.99 [0.43 ; 2.3] | 0.99 | 0.99 [0.43 ; 2.3] | 0.99 |  |  |
| **Seizure** | 1.02 [0.27 ; 3.89] | 0.98 | 1.02 [0.27 ; 3.89] | 0.98 |  |  |
| **Status epilepticus** | 3.30 [0.41 ; 26.53] | 0.26 | 3.30 [0.41 ; 26.53] | 0.26 |  |  |
| **Presence of sedation** | 0.52 [0.21 ; 1.25] | 0.14 | 0.52 [0.21 ; 1.25] | 0.14 | 0.68 [0.41 ; 1.13] | 0.14 |

Results are expressed as OR [95 CI]. Abbreviations: AI: after imputation (multiple imputations were used to adjust for missing values), BI: before imputation, Hz: Hertz, LPD: Lateralized periodic discharges

**Supplementary Table 4:** Complete case analysis for the whole cohort (n = 194), and among patients under mechanical ventilation (n = 122)

| **Whole cohort (n = 194)** | | |
| --- | --- | --- |
| **Variables** | **OR [95CI]** | **p-value** |
| **Age** |  |  |
| ≥ 50 and < 65 | 3.2 [1.24; 8.29] | 0.57 |
| ≥ 65 and < 75 | 1.87 [0.67; 5.21] | 0.26 |
| ≥ 75 | 8.98 [2.36; 34.17] | 0.01 |
| < 50 | 1 | <0.01 |
| **Glasgow coma scale < 8**, indicating coma | 1.35 [0.6; 3.02] | 0.46 |
| **Temperature ≥ 38.3°C,** indicating fever | 2.04 [0.94; 4.41] | 0.07 |
| **Time between ICU admission and _std_EEG** **> 1 day** | 2.84 [1.19; 6.77] | 0.02 |
| < 7Hz | 1.8 [0.77; 4.2] | 0.74 |
| Background frequency not specified | 2.49 [0.85; 7.31] | 0.21 |
| Alpha (> 7Hz) | 1 | 0.20 |
| **Reactivity** |  |  |
| Absent | 2.71 [1.03; 7.12] | 0.02 |
| Not tested | 0.93 [0.38; 2.27] | 0.16 |
| Present | 1 | 0.06 |
| **Sedation** | 0.76 [0.34; 1.67] | 0.49 |
| **Patients under mechanical ventilation (n = 122)** | | |
| **Age** |  |  |
| ≥ 50 and < 65 | 4.08 [0.95; 17.45] | 0.57 |
| ≥ 65 and < 75 | 3.84 [0.8; 18.37] | 0.71 |
| ≥ 75 | 6.95 [1.26; 38.17] | 0.16 |
| < 50 | 1 | 0.12 |
| **Glasgow coma scale < 8**, indicating coma | 0.97 [0.34; 2.79] | 0.96 |
| **Temperature ≥ 38.3°C**, indicating fever | 2.82 [0.92; 8.62] | 0.07 |
| **Time between ICU admission and _std_EEG > 1 day** | 8.81 [1.93; 40.15] | <0.01 |
| **Reactivity** |  |  |
| Absent | 6.84 [1.82; 25.72] | 0.04 |
| Not tested | 3.82 [1.05; 13.92] | 0.51 |
| Present | 1 | 0.01 |
| **Presence of sedation** | 0.37 [0.12; 1.13] | 0.08 |

Abbreviations: ICU: intensive care unit, _std_EEG: standard electroencephalography
